# Real-World Comparison of Intravenous vs. Oral Antimicrobial Therapy for Bone and Joint Infections

**DOI:** 10.3390/pharmacy14020048

**Published:** 2026-03-14

**Authors:** Maura Kreiser, Sarah Al Mansi, Ismaeel Yunusa, Caroline Derrick, P. Brandon Bookstaver, Majdi N. Al-Hasan, Yorika Hammett, Morgan Pizzuti

**Affiliations:** 1 Prisma Health Richland, Columbia, SC 29203, USA; caroline.derrick@prismahealth.org (C.D.); bookstaver@cop.sc.edu (P.B.B.); majdinaser.alhasan@prismahealth.org (M.N.A.-H.); morgan.pizzuti@prismahealth.org (M.P.); 2 Prisma Health Tuomey, Sumter, SC 29150, USA; sarah.almansi@prismahealth.org (S.A.M.);; 3 Department of Clinical Pharmacy and Outcomes Sciences, College of Pharmacy, University of South Carolina, Columbia, SC 29208, USA; iyunusa@mailbox.sc.edu; 4 School of Medicine, University of South Carolina, Columbia, SC 29203, USA

**Keywords:** osteomyelitis, septic arthritis, spinal infections, *Staphylococcus aureus*

## Abstract

Well-designed randomized controlled trials (RCTs) have demonstrated safe and effective use of oral antimicrobial therapy for bone and joint infections. Application of data for implementation into real-world practice, however, has inherent challenges. This retrospective cohort study compared real-world use of intravenous versus oral antimicrobial therapy in bone and joint infections within a large healthcare system comprising both academic and community medical centers. The primary outcome was the proportion of treatment failure. Key secondary outcomes included the proportion of patients with logistical failure and risk factors associated with treatment and logistical failure. Among 166 patients included, 136 (82%) and 30 (18%) received predominantly intravenous and oral antimicrobial therapy, respectively. Treatment failure occurred in (77/121) 64% versus (18/25) 72% of patients in the intravenous and oral antimicrobial groups (*p* = 0.491; OR, 1.38; 95% CI, 0.56–3.33). Logistical failure occurred in 29% versus 47% of patients in the intravenous and oral antimicrobial groups (*p* = 0.150; OR, 1.93; 95% CI 0.79–4.70). Risk factors for treatment failure included peripheral vascular disease (OR, 2.61; 95% CI 1.02–7.80) and higher Charlson Comorbidity Index scores (OR, 1.18; 95% CI 1.04–1.36). Similar to previously published RCTs, treatment failure appeared comparable between groups; however, oral antimicrobial therapy was overall underutilized.

## 1. Introduction

Outpatient Parenteral Antimicrobial Therapy (OPAT) has seen significant growth over the past few decades, driven by clinical efficiency and cost-effectiveness. The primary goal of OPAT is to allow patients to complete antimicrobial treatment safely and effectively in the comfort of their own home or at another outpatient site [1]. However, a systematic review and meta-analysis comparing OPAT to inpatient treatment demonstrated additional benefits, including a 36% reduction in treatment-related costs [2,3]. This approach has long become a standard of care for various infections, including bone and joint infections, which often require protracted courses of antimicrobial therapy. More recently, numerous well-designed, randomized controlled trials (RCTs), including pivotal ones, such as OVIVA (Oral versus Intravenous Antibiotics for Bone and Joint Infection), have demonstrated comparable effectiveness and safety of oral and intravenous antimicrobial regimens for the treatment of bone and joint infections, challenging the traditional preference for prolonged intravenous antimicrobial courses [4,5]. A shift to oral antimicrobial regimens is also accompanied by several advantages for the healthcare system and patients, including lower overall treatment costs, improved patient satisfaction, and reduced invasive intravenous line days, thus avoiding line-related complications [6,7,8,9,10,11,12]. Direct application of these RCTs to clinical practice, however, can be limited and challenge clinicians who are seeking to adhere to evidence-based practice. Often, the inclusion criteria and/or study sites (e.g., large urban versus rural) in RCTs do not match the local patient case mix. Additionally, oral regimens utilized in the majority of RCTs may not match the preferred regimens of clinicians who favor highly bioavailable regimens with infrequent dosing to improve adherence. Availability of alternative long-acting treatments (e.g., dalbavancin or oritavancin) has also influenced patient selection for both OPAT and oral-based regimens. A lack of outcome data on the real-world implementation of commonly prescribed oral regimens for deep-seated bone and joint infections remains, particularly in healthcare systems across the Southeastern United States.

This retrospective cohort study compares clinical and logistical outcomes of oral versus intravenous antimicrobial regimens in the treatment of bone and joint infections within health systems in South Carolina, a region with socioeconomic diversity.

## 2. Materials and Methods

This observational, retrospective cohort study was conducted within four hospitals in the Prisma Health Midlands system, comprising both community and teaching hospitals. The study was approved by the Institutional Review Board, with a waiver of informed consent. Eligible patients were adults admitted to and seen by an infectious diseases provider and later discharged from one of the study sites between January and August 2023. The institution has a robust multidisciplinary OPAT team, including two dedicated nurses, one dedicated advanced practice provider, fifteen physicians, and three pharmacists. The study population included patients with documented or presumed bacterial bone and/or joint infections, defined as native osteomyelitis (of long bones, small bones, or spine), native joint infection, prosthetic joint infection, orthopedic fixation-device infection, and/or epidural abscess, with a planned antimicrobial treatment duration of at least four weeks. Patients were excluded if they received two weeks or less of antimicrobial therapy, received primary treatment for severe nontuberculous mycobacterial, fungal, or viral infections, completed antimicrobial therapy in the hospital, had an expected treatment duration longer than 6 months, or were incarcerated.

Antimicrobial therapy was selected by an infectious diseases provider, and patients were categorized into oral or intravenous antimicrobial groups based on the route of administration for the majority of their total treatment duration. Those who received intravenous antimicrobials for >50% of treatment days were assigned to the intravenous group, while those who received oral antimicrobials for >50% of treatment days were assigned to the oral group. The intention-to-treat (ITT) population included all eligible patients. The per-protocol (PP) population was restricted to patients with complete documentation throughout the one-year follow-up period, defined as the presence of both an inpatient sign-off note and a corresponding outpatient follow-up note.

The primary endpoint was the proportion of patients experiencing treatment failure and was analyzed in the PP population. Treatment failure was defined as meeting one or more of the following criteria: provider-determined failure (i.e., extension of treatment beyond planned duration), microbiological recurrence of the same pathogen (from tissue, bone, or blood cultures), readmission for suspected infection progression, radiologic or operative evidence of persistent infection, all-cause mortality, or discontinuation or change in therapy due to adverse effects or intolerance. Additionally, a post hoc analysis of treatment failure in the PP population was performed, excluding provider-assessed failure at follow-up, all-cause mortality, and discontinuation or change in therapy due to adverse events or intolerance from treatment failure criteria to more closely align with endpoints in the OVIVA trial.

Secondary endpoints were analyzed in the ITT population and included the proportion of patients with logistical failure, defined as meeting one or more of the following: documented non-adherence to therapy, barriers to medication access, intravenous line complications, or failure to complete appropriate follow-up (defined as attending ≥ 1 infectious disease follow-up visit). Additional secondary outcomes included the incidence of treatment-emergent adverse events (TEAEs) and identification of risk factors for treatment and logistical failure.

### Statistical Analysis

Data were collected for up to one year following initiation of antimicrobial therapy. Descriptive statistics summarized baseline characteristics, proportion of treatment failure, proportion of logistical failure, and proportion of TEAE stratified by treatment route. Categorical variables were compared using chi-square or Fisher’s exact tests, as appropriate, and continuous variables were compared using an unpaired *t* test. A two-sided *p*-value of <0.05 was considered statistically significant. All estimates are reported with 95% confidence intervals (CIs).

Univariable (unadjusted) Firth logistic regression analyses were performed to identify potential risk factors of treatment and logistical failure. Variables that were deemed clinically important or showed *p* < 0.20 in these analyses were included in the multivariable model. Backward elimination was then applied to derive the final adjusted model while retaining variables with meaningful clinical or statistical contributions. Firth’s penalized logistic regression was used for both univariable and multivariable analyses to reduce small-sample bias and generate stable parameter estimates when event numbers were low or unevenly distributed. This approach was chosen because it prevents unreliable or infinite ORs that can occur with conventional logistic regression when data are sparse [13]. Adjusted ORs with 95% CIs and two-sided *p*-values are reported.

Model diagnostics included assessment of multicollinearity to ensure predictors were not highly correlated, which can distort effect estimates, and evaluation of overall model fit to confirm that the model adequately represented the observed data. These steps were performed to enhance confidence that the final models were both statistically valid and clinically interpretable. All analyses were conducted using R version 4.3.2 (R Foundation for Statistical Computing, Vienna, Austria).

## 3. Results

**Figure 1:**
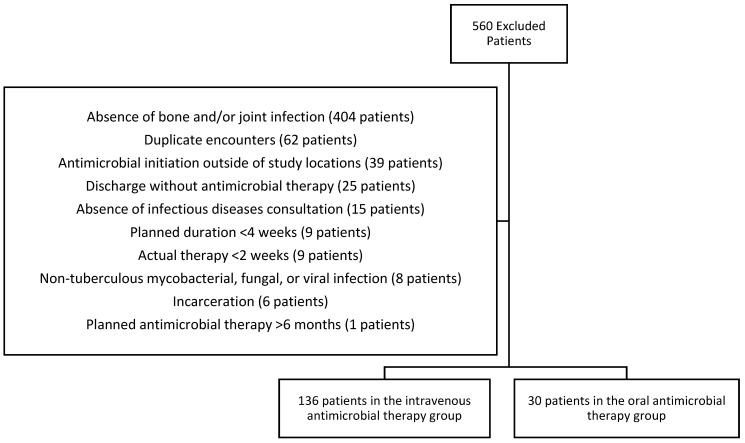
Exclusion criteria met. Eleven patients met two exclusion criteria, 2 patients met three, and 1 patient met four.

A total of 726 patients were screened, 166 met the inclusion criteria and were included in the analysis, and 560 patients were excluded outlined in Figure 1. Of the 166 patients included in the analysis, 136 were assigned to the intravenous antimicrobial group and 30 to the oral antimicrobial group in the intention-to-treat (ITT) population. Due to loss of follow-up, the per-protocol analysis included 146 patients—121 in the intravenous group and 25 in the oral group.

Baseline characteristics are summarized in Table 1. The majority of patients received treatment at home, followed by administration at a facility. Most infections involved native bones and joints. However, hardware involvement was documented in 19 patients (14%) in the intravenous antimicrobial group and 5 patients (17%) in the oral antimicrobial group (Table 1). Osteomyelitis was the most commonly treated infection, with the toe/foot being the most common anatomical location. Diabetic foot infections involving osteomyelitis of the foot and/or toe were present in 37 intravenous antimicrobial group patients (27%) and 12 oral antimicrobial group patients (40%).

Microbiological cultures demonstrated polymicrobial growth in 44 patients (32%) in the intravenous antimicrobial group and 14 patients (47%) in the oral antimicrobial group. Cultures were either unavailable or showed no growth in 39 patients in the intravenous antimicrobial group (29%) and 7 patients in the oral antimicrobial group (23%). *Staphylococcus aureus* was most frequently cultured in monomicrobial settings (Table 2). In the intravenous antimicrobial group, the most commonly used intravenous antimicrobials were vancomycin (75 patients, 55%) and cephalosporins (113 patients, 83%). In the oral group, fluoroquinolones (17 patients, 57%) and aminopenicillins (9 patients, 30%) were the most frequently used antimicrobial classes (Table 3). The mean length of antimicrobial therapy determined by the provider was 46.6 days in the intravenous antimicrobial group and 54.8 days in the oral antimicrobial group.

The primary endpoint of treatment failure occurred in 77 patients in the intravenous antimicrobial group (64%) and 18 patients in the oral antimicrobial group (72%). This difference was not statistically significant (*p* = 0.43). Table 4 summarizes the criteria met for treatment failure. The most common criteria were provider-determined treatment failure at follow-up office visits and hospital readmission due to suspected worsening of infection. Of the 41 patients who experienced provider-determined treatment failure, all received an extension of antimicrobial therapy. Nine of these patients were noted to have non-healing wounds or wound dehiscence, five patients were noted to have signs and symptoms of infection at the time of follow-up, three patients had imaging findings leading to antimicrobial extension, three patients had underdosed antimicrobial therapy, one patient had non-adherence to antimicrobial therapy due to hospitalization, and the remaining twenty patients had subjective changes in the therapy plan based on provider preference. The post hoc analysis, with the exclusion of provider-assessed failure at follow-up, all-cause mortality, and discontinuation or change in therapy due to adverse events or intolerance from treatment failure criteria, showed treatment failure rates were 30% (36/121 patients) in the intravenous antimicrobial group vs. 40% (10/25 patients) in the oral antimicrobial group.

Logistical failure was observed in 39 patients in the intravenous antimicrobial group (29%) and 14 patients in the oral antimicrobial group (47%) (*p* = 0.056). The most common criteria met for logistical failure in both the intravenous and oral antimicrobial groups were missing all recommended follow-up visits in the infectious disease clinic (Table 5). There were no statistically significant differences in TEAE between the groups (Table 6). Thrombocytopenia was the most common TEAE in the intravenous antimicrobial group (20 patients, 15%), and rash was the most common TEAE in the oral antimicrobial group (4 patients, 13%). Peripheral vascular disease (PVD) was associated with increased risk of treatment failure (odds ratio [OR] 2.80; 95% confidence intervals [CI] 1.06–2.74; *p* = 0.05). Higher Charlson Comorbidity Index scores were also associated with increased risk of treatment failure (OR 1.18; 95% CI 1.04–1.36). No significant risk factors were identified for logistical failure (Table 7).

## 4. Discussion

This study resulted in 64% treatment failure in the intravenous antimicrobial group vs. 72% treatment failure in the oral antimicrobial group (*p* = 0.43). This treatment difference was larger than previously reported in the literature due to broader, more inclusive criteria used to define treatment failure in this study. While OVIVA defined failure using specific clinical, microbiologic, or histologic parameters, this study incorporated real-world clinical decision-making, leading to higher treatment failure rates in this study compared to OVIVA (64% vs. 14.6% in the intravenous antimicrobial group; 72% vs. 13.2% in the oral antimicrobial group). Treatment failure in this study was defined not only by clinical signs of infection but also by provider-assessed failure at follow-up, all-cause mortality, and discontinuation or change in therapy due to adverse events or intolerance. When these additional criteria were excluded, treatment failure rates were notably lower (30% (n = 36) patients in the intravenous antimicrobial group vs. 40% (n = 10) patients in the oral antimicrobial group), more closely aligning with the existing literature. The provider assessment of treatment failure at follow-up criterion alone made up a large portion of treatment failure, accounting for 26% of patients with treatment failure in the intravenous antimicrobial group and 36% of patients with treatment failure in the oral antimicrobial group. Though this captures real-world practice, it may be a limitation since it is subjective and at the discretion of the individual provider at the ID clinic follow-up visit, which may differ from the provider who created the treatment plan in the hospital.

To more accurately replicate a real-world patient population, in addition to broader inclusion criteria, the exclusion criteria in this study were less limiting. Two key exclusion criteria in the OVIVA trial that were allowed in this cohort study and likely played a role in the difference in failure rates were *S. aureus* bacteremia on presentation or within the past month and the patient’s unlikelihood to comply with trial requirements following randomization. First, *S. aureus* bacteremia is known to have poor outcomes, and this study included 21 patients (13%) who had a blood culture positive with *S. aureus* (20/21 patients in the intravenous antimicrobial group, 1/21 patients in the oral antimicrobial group). Mortality was a component of treatment failure in this study, and Bai et al. performed a systematic review and meta-analysis that described 536,791 patients and estimated high mortality rates, up to 30.2% at 1 year [14]. Additionally, the inclusion of patients who were unlikely to comply with the requirements in this study made the population more reflective of real-world practice.

Azamgarhi et al. looked at treatment failure rates in bone and joint infections post-implementation of the OVIVA trial, with a similar sample size to this study (166 patients versus 183 patients post-OVIVA study protocol implementation). Treatment failure rates were also higher in this study compared to Azamgarhi et al. (64.0% vs. 26.7% in the intravenous antimicrobial group; 72.0% vs. 14.3% in the oral antimicrobial group) because the criteria for treatment failure in Azamgarhi et al. mirrored the OVIVA study protocol. Though logistical failure was not a study endpoint, individual components of the criteria for logistical failure were reported. The results of this study compared to the results of Azamgarhi et al. were as follows: loss to follow-up (20% versus 0% in the intravenous antimicrobial group and 30% versus 0% in the oral antimicrobial group), discontinuation or change in therapy (16 patients versus 6 patients in the intravenous antimicrobial group and 5 patients vs. 21 patients in the oral antimicrobial group), and line complications in the intravenous antimicrobial group (13% incidence versus three complications/1000 line-days) [15].

The assessment of logistical failure as a composite endpoint was a novel aspect of this study and is an important consideration in real-world antimicrobial stewardship. The higher rate of logistical failure (47% vs. 29%, *p* = 0.056) in the oral antimicrobial group may reflect challenges such as medication adherence, access to follow-up care, and socioeconomic factors. For example, a large portion (23%) of the oral antimicrobial group received linezolid at hospital discharge, and it is often a non-formulary medication requiring prior insurance authorization. This aligns with our data, with 23% of patients who experienced logistical failure having barriers to medication access. Though intravenous antimicrobial agents are also high in cost, there are more dedicated multidisciplinary team members (nurses, case management, etc.) at our institution that coordinate with home infusion and insurance companies before patients are discharged from the hospital with intravenous agents. Additionally, patients in the intravenous antimicrobial group receive extensive counseling on antimicrobial administration and a dedicated nurse visit with a representative from the infusion company, compared to patients in the oral antimicrobial group. This is likely associated with the noncompliance rates of 3% of patients in the intravenous antimicrobial group versus 13% of patients in the oral antimicrobial group. These findings may have been influenced by selection bias inherent to the retrospective design. Social determinants of health likely impacted treatment selection, with patients facing greater logistical or financial barriers more frequently prescribed oral regimens. For example, 16 (10%) patients did not have insurance coverage in this study, impacting treatment selection and disposition. Additionally, institutional protocols for patients receiving OPAT versus complex outpatient antimicrobial therapy (COpAT) involve more structured monitoring, which may have led to better oversight and early identification of complications in the intravenous group. Patients who receive OPAT are monitored with weekly laboratory testing and chart review by one of the healthcare providers, while patients who receive COpAT are monitored with every 2–6 weeks of laboratory testing based on the provider’s preference and monthly chart review by one of the OPAT team members.

Two key antimicrobial agents utilized in bone and joint infections that emerged since the OVIVA trial was performed are dalbavancin and linezolid. The literature has shown that loss to follow-up is a barrier to using dalbavancin. In this study, 12 patients (7%) received dalbavancin as part of their treatment regimen, and 5 of these patients experienced treatment failure (42%). Two of these failures were patients who were lost to follow-up and could not be evaluated (17%). These results are comparable to those published in the current literature [16,17,18]. Crocker et al. reported 29.4% (57/194 patients) clinical failure with dalbavancin, with loss to follow-up being a large driver of clinical failure (30/194 patients, 15.5%) [16]. Lovatti et al. also reported criteria met for treatment failure, with 1.2% of patients meeting criteria for loss to follow-up [18]. These high rates of loss to follow-up show that even robust OPAT programs cannot replace the need for patient ownership, especially with long-acting agents such as dalbavancin.

Additionally, 14 patients (8%) received intravenous or oral linezolid as part of their treatment regimen, and 11 of these patients experienced treatment failure (78.5%). One barrier to linezolid use is prescriber hesitation in infections with prosthetic material because of its bacteriostatic activity. However, of the 11 patients who experienced treatment failure, 1 had an infection involving prosthetic material, and 2/3 patients who did not experience treatment failure had an infection involving prosthetic material. Studies reviewed linezolid and reported overall success rates. Morata et al. reported a 79.9% remission rate at three-month follow-up in orthopedic implant infections. Theil et al. reported 80% of patients achieving infection control in periprosthetic joint infections, Senneville et al. reported 78.8% overall successful cure in chronic osteomyelitis, and Aneziokoro et al. reported 55% clinical cure in chronic osteomyelitis [19,20,21,22]. The other barrier to linezolid use is prescriber hesitancy due to bone marrow suppression. Veerman et al. examined prolonged use of linezolid and reported 10% of patients developing a cytopenia defined as a hemoglobin level < 4.9 mmol/L, a white blood cell count < 4.0 × 10^9^/L, or a platelet count < 75 × 10^9^/L [23]. Comparatively, there were no patients in this study who developed a hemoglobin level < 4.9 mmol/L or a platelet count < 75 × 10^9^/L. However, only nine patients had these values monitored while on linezolid therapy, and of the nine patients, two received linezolid for less than 10 days.

Despite these limitations, a strength of the study lies in the thoughtful selection of oral antimicrobial regimens. All patients in the oral antimicrobial group received agents consistent with guideline-recommended options for musculoskeletal infections, including high-bioavailability antimicrobials with favorable bone penetration [24]. Thabit et al. reviewed antibiotic penetration into bone, defining good penetration as concentrations exceeding the MIC_90_ and/or MIC breakpoints of common bone and joint pathogens [25]. All patients in the oral antimicrobial group of this study received at least one of the agents defined as having good penetration as definitive therapy. An area for improvement is combination therapy with rifampin. In the OVIVA trial, 48.2% of patients received rifampicin; however, in this cohort, there were only two (1%) patients who received rifampin combination regimens. Although this highlights an opportunity to optimize therapy, it also reflects the real-world challenges associated with rifampin use, including its significant drug–drug interactions and tolerability.

The emergence of peripheral vascular disease as a risk factor was particularly relevant in this real-world cohort based on location. All health systems included are located in the state of South Carolina, where the reported rate of diabetes diagnosis is approximately 13.5% of the adult population, and prediabetes diagnosis is approximately 34.9% of the adult population [26]. The zip code of the primary site of this study had a rate of lower limb amputations related to diabetes 1.8 times higher than the state rate between 2019 and 2023 [27]. Overall, nearly one-third of the cohort had a diabetic foot infection, and 13% had both diabetes and peripheral arterial disease (PVD). Given that PVD is strongly associated with impaired wound healing, recurrent infection, amputation, and mortality in patients with diabetes, it was not surprising that PVD emerged as a risk factor for treatment failure [28,29,30,31,32]. A recent study by Hawkins et al. also showed that 7 of the 11 patients who experienced treatment failure when using oral antimicrobial therapy for bone and/or joint infections had both PVD and diabetes [33].

In addition to selection bias based on social determinants of health and differing institutional protocols for OPAT versus COpAT discussed previously, limitations of this study included the short follow-up time and relatively small sample size in the oral antimicrobial group. Given the chronic nature of bone and joint infections, a one-year follow-up period may be insufficient to fully capture long-term outcomes. Additionally, despite data showing no differences in rates of treatment failure, hesitancy remains when treating patients with oral antibiotics for bone and joint infections due to numerical differences in other factors, such as hospital readmission rates. For example, 40% of patients in the oral group in this study were readmitted due to suspected worsening infection compared to only 25% in the intravenous group. Therefore, there was an uneven distribution of patients included in the intravenous versus oral antimicrobial group due to the observational design.

There were also low rates of patients with documented source control in the intravenous antimicrobial group. A total of 61% of patients in the intravenous antimicrobial group received source control compared to 77% in the oral antimicrobial group. Since source control is essential for the successful treatment of bone and joint infections, the lower rate of source control in the intravenous group may have contributed to poorer outcomes. Selection bias may have been introduced by restricting inclusion to patients seen by an infectious disease provider. This criterion captures most patients with a bone and/or joint infection at the study institutions and almost all the patients who received intravenous antibiotics at discharge based on practices at the study institution. However, there may have been patients treated with oral antimicrobial agents for bone and/or joint infections that were not included in the study with this design. Patients who were lost to follow-up were excluded from the primary endpoint analysis. If these patients differed systematically from those with complete follow-up, this may have introduced attrition bias and could contribute to immortal time bias. Finally, inherent to the retrospective design, treatment assignment was unblinded, which may have introduced outcome ascertainment bias.

This data showed underutilization of oral antimicrobial agents in real-world practice. Eighteen (11%) patients in this study experienced PICC line complications. Additionally, IV antimicrobial therapy has an inherent burden on the healthcare system, requiring dedicated nursing time, infusion resources, vascular access maintenance, and clinical monitoring, all contributing to higher costs and increased workflow complexity. The institutions where this study was performed do not have local guidelines or protocols to direct treatment durations or preferred oral regimens. The decision was subjective and up to the personal preference and discretion of the individual provider. After the OVIVA trial, Hawkins et al. implemented guidelines to preferentially treat patients with bone and joint infections with oral antibiotics. After guideline implementation, the difference in patients who were more likely to be discharged exclusively on oral antibiotics was reported at 45% (25 vs. 70% pre-implementation vs. post-implementation) [33]. The direct impact on the utilization of oral antimicrobial agents suggests this strategy could improve usage of oral antimicrobial therapy in real-world practice.

## 5. Conclusions

The difference in cohort sizes in this study demonstrated the underutilization of oral antimicrobial therapy in real-world practice despite the previous literature that supports the use of oral antimicrobial therapy for bone and/or joint infections. Though the sample size was small, this study adds to the existing literature, highlighting the importance of considering both clinical and logistical factors in real-world antimicrobial decision-making. A crucial component for treatment success is to proactively address logistical challenges in these patients who require protracted courses of antibiotics.

## Figures and Tables

**Table 1:** Baseline clinical characteristics of patients with bone and/or joint infections in the intention-to-treat population.

	Intention-to-Treat Population			Per Protocol Population		
Characteristic	Intravenous Antimicrobial Group (*n* = 136)	Oral Antimicrobial Group (*n* = 30)	*p*-Value	Intravenous Antimicrobial Group (*n* = 121)	Oral Antimicrobial Group (*n* = 25)	*p*-Value
Age, in years, mean (SD)	60 (15)	58 (16)	0.51	60 (15)	59 (16)	0.76
Male sex, *n* (%)	76 (56)	19 (63)	0.56	66 (55)	17 (68)	0.22
Charlson Comorbidity Index, points, median (IQR)	4 (2–6)	3 (2–4)	0.06	4 (2–6)	3 (2–4)	0.13
Peripheral vascular disease, *n* (%)	19 (14)	6 (20)	0.40	18 (15)	4 (16)	1.00
Liver disease, *n* (%)	9 (7)	1 (3)	0.69	7 (6)	1 (4)	1.00
Diabetes mellitus, *n* (%)	71 (52)	15 (50)	0.83	64 (53)	13 (52)	0.94
HbA1c > 7, *n* (%)	40 (29)	11 (37)		36 (30)	9 (36)	
Chronic kidney disease, *n* (%)	15 (11)	0 (0)	0.08	12 (10)	0 (0)	0.22
Immunocompromised host *, *n* (%)	20 (15)	0 (0)	0.03	17 (14)	0 (0)	0.04
Intravenous drug use, *n* (%)	6 (4)	2 (7)	0.64	4 (3)	1 (4)	1.00
Length of hospital stay, in days, median (IQR)	11 (7–19)	6 (4–11)	0.30	11 (7–18)	6 (5–11)	0.41
Discharge location						
Home, *n* (%)	86 (63)	24 (80)	0.08	77 (64)	19 (76)	0.24
Facility, *n* (%)	50 (37)	6 (20)		44 (37)	6 (24)	
Insurance						
Medicaid, *n* (%)	70 (51)	19 (63)	0.24	66 (55)	16 (64)	0.39
Medicare, *n* (%)	18 (13)	4 (13)	1.00	14 (12)	3 (12)	1.00
BCBS, *n* (%)	17 (13)	2 (7)	0.53	16 (13)	1 (4)	0.31
Uninsured, *n* (%)	11 (8)	5 (17)	0.15	7 (6)	5 (20)	0.02
Other, *n* (%)	20 (15)	0 (0)	0.03	18 (15)	0 (0)	0.04
Source control, *n* (%)	83 (61)	23 (77)	0.11	72 (60)	21 (84)	0.02
Suppressive antimicrobial therapy on admission, *n* (%)	3 (2)	1 (3)	0.55	3 (2)	1 (4)	0.53
Administration Location ^						
Home, *n* (%)	70 (51)	23 (77)	0.01	65 (54)	18 (72)	0.09
Facility, *n* (%)	50 (37)	6 (20)	0.08	44 (36)	6 (24)	0.23
Hemodialysis center, *n* (%)	8 (6)	1 (3)	1.00	7 (6)	0 (0)	0.6
Infusion center, *n* (%)	8 (6)	0 (0)	0.35	5 (4)	1 (4)	1.00
Infection Type ǂ						
Native bone osteomyelitis,						
*n* (%)	82 (60)	20 (67)	0.52	73 (60)	17 (68)	0.51
Spinal infection, *n* (%)	33 (24)	4 (13)	0.23	30 (25)	3 (12)	0.20
Orthopedic fixation						
device infection, *n* (%)	17 (13)	3 (10)	1.00	15 (12)	3 (12)	1.00
Native joint infection, *n* (%)	16 (12)	4 (13)	0.76	13 (11)	4 (16)	0.49
Prosthetic joint infection, *n* (%)	2 (1)	2 (7)	0.15	2 (2)	1 (4)	0.43

BCBS = Blue Cross Blue Shield; * Immunocompromised patients included patients with a solid tumor, leukemia, lymphoma, or AIDS. ^ In the intravenous antimicrobial group, 10 patients who received antimicrobials at home also received oral antimicrobials; however, they did not meet the criteria to be included in the oral group due to the duration of oral therapy. In the oral antimicrobial group, seven patients who received antimicrobials at home and one patient who received antimicrobials at the infusion center were on intravenous antimicrobials; however, they did not meet the criteria to be included in the intravenous group due to the duration of intravenous therapy. ǂ Fourteen patients had multiple infection types in the intravenous antimicrobial group, and three patients had multiple infection types in the oral antimicrobial group.

**Table 2:** Microbiology of isolates with monomicrobial growth in the ITT population.

Organism	Intravenous Antimicrobial Group (*n* = 136)	Oral Antimicrobial Group (*n* = 30)	*p*-Value
*Staphylococcus aureus*, *n* (%)	25 (18)	2 (7)	0.17
MSSA	18 (13)	2 (7)	0.53
MRSA	7 (5)	0 (0)	0.35
*Streptococcus* spp., *n* (%)	10 (7)	1 (3)	0.69
Enterobacterales, *n* (%)	7 (5)	3 (10)	0.39
*Pseudomonas aeruginosa*, *n* (%)	1 (0.7)	1 (3)	0.33
Obligate anaerobic bacteria, *n* (%)	1 (0.7)	2 (7)	0.08
Other bacteria *, *n* (%)	3 (2)	0 (0)	1.00

This table is representative of isolates with monomicrobial growth only. Additionally, there were 44 (32%) patients in the intravenous antimicrobial group and 14 (47%) patients in the oral antimicrobial group who had isolates with polymicrobial growth. The remaining patients did not have cultures with microbial growth. MSSA = methicillin-susceptible *Staphylococcus aureus* and MRSA = methicillin-resistant *Staphylococcus aureus* * Other bacteria included *Cutibacterium* spp. and *Acinetobacter* spp.

**Table 3:** Antimicrobial agents in the intention-to-treat population.

Agent, n (%)	Intravenous Antimicrobial Group (*n* = 136)	Oral Antimicrobial Group (*n* = 30)	*p*-Value
**Intravenous**			
Cephalosporins	113 (83)	16 (53)	0.0004
Cefepime	48 (35)	11 (37)	0.89
Ceftriaxone	65 (48)	5 (17)	0.002
Cefazolin	22 (16)	1 (3)	0.08
Ceftaroline	4 (3)	0 (0)	1.00
Ceftazidime	1 (1)	0 (0)	1.00
Vancomycin	75 (55)	14 (47)	0.40
Daptomycin	57 (42)	4 (13)	0.08
Penicillins	23 (17)	7 (23)	0.16
Piperacillin/tazobactam	9 (7)	3 (10)	0.46
Ampicillin/sulbactam	8 (6)	3 (10)	0.42
Penicillin	4 (3)	1 (3)	1.00
Ampicillin	2 (1)	0 (0)	1.00
Carbapenems	18 (13)	3 (10)	0.77
Metronidazole	15 (11)	3 (10)	1.00
Dalbavancin	12 (9)	1 (3)	0.47
Linezolid	4 (3)	0 (0)	1.00
Fluoroquinolones	2 (1)	1 (3)	0.45
Ciprofloxacin	1 (1)	1 (3)	0.33
Levofloxacin	1 (1)	0 (0)	1.00
Aminoglycosides	2 (1)	0 (0)	1.00
Clindamycin	1 (1)	0 (0)	1.00
**Oral**			
Metronidazole	50 (37)	8 (27)	0.29
Fluoroquinolones	20 (15)	17 (57)	0.0001
Levofloxacin	14 (10)	10 (33)	0.001
Ciprofloxacin	6 (4)	7 (23)	0.0005
Penicillins	8 (6)	9 (30)	0.0001
Amoxicillin/clavulanate	7 (5)	6 (20)	0.006
Amoxicillin	1 (1)	4 (13)	0.004
Linezolid *	7 (5)	7 (23)	0.001
Doxycycline	5 (4)	3 (10)	0.16
Clindamycin	2 (1)	0 (0)	1.00
Rifampin	2 (1)	0 (0)	1.00
Cephalexin	1 (1)	5 (17)	0.0008
Sulfamethoxazole/trimethoprim	1 (1)	2 (7)	0.08

Agents were captured starting from the infectious diseases provider-determined start date; therefore, empiric therapy may not be included. Patients were on multiple antimicrobial agents throughout their treatment course, including both intravenous and oral agents, and agents in the same drug class. Bolded headings are used to represent antimicrobial route categories. * One patient in the intravenous antimicrobial group received tedizolid, and one patient in the oral antimicrobial group received tedizolid. The remaining patients received linezolid.

**Table 4:** Criteria met for treatment failure in the PP population. The intravenous antimicrobial group had 15 patients who were not included due to loss to follow-up, and the oral antimicrobial group had 5 patients who were not included due to loss to follow-up. The intravenous antimicrobial group had 41 patients with multiple reasons for treatment failure, and the oral antimicrobial group had 11 patients with multiple reasons for treatment failure.

Criteria Met for Treatment Failure	Intravenous Antimicrobial Group (*n* = 121)	Oral Antimicrobial Group (*n* = 25)	*p*-Value
Provider-determined treatment failure at follow-up office visit, *n* (%)	32 (26)	9 (36)	0.33
Hospital readmission due to suspected worsening of infection, *n* (%)	30 (25)	10 (40)	0.12
Radiologic or operative findings suggestive of ongoing infection, *n* (%)	29 (24)	9 (36)	0.21
Mortality, *n* (%)	20 (17)	1 (4)	0.13
Discontinuation of agents or use of alternative antimicrobial agents due to adverse event/intolerability, *n* (%)	16 (13)	5 (20)	0.38
Microbiological recurrence, *n* (%)	8 (7)	3 (12)	0.40

**Table 5:** Criteria met for logistical failure in the intention-to-treat population.

Criteria Met for Logistic Failure	Intravenous Antimicrobial Group (*n* = 136)	Oral Antimicrobial Group (*n* = 30)	*p*-Value
Missed all recommended follow-up visits with ID, *n* (%)	27 (20)	9 (30)	0.22
PICC line complication, *n* (%)	17 (13)	1 (3) *	0.20
Medication access barrier, *n* (%)	13 (10)	7 (23)	0.04
Non-adherence documented, *n* (%)	4 (3)	4 (13)	0.04

* Some patients in the oral antimicrobial group received a PICC line, as classification into this group was based on receiving oral therapy for more than 50% of total treatment days.

**Table 6:** Treatment-emergent adverse events in the intention-to-treat population.

TEAE	Intravenous Antimicrobial Group (*n* = 136)	Oral Antimicrobial Group (*n* = 30)	*p*-Value
Overall	44 (32)	9 (30)	0.80
Thrombocytopenia, *n* (%) ^	20 (15)	3 (10)	0.77
AKI, *n* (%)	12 (9)	2 (7)	1.00
Rash, *n* (%)	5 (4)	4 (13)	0.06
Transaminitis, *n* (%)	4 (3)	1 (3)	1.00
Neurologic effects, *n* (%)	4 (3)	1 (3)	1.00
Eosinophilic Pneumonia, *n* (%)	3 (2)	1 (3)	0.55
Diarrhea, *n* (%)	3 (2)	1 (3)	0.55
Rhabdomyolysis, *n* (%) *	2 (1)	0	1.00

* Two additional patients had abnormalities with CK in the intravenous group with no rhabdomyolysis; ^ thrombocytopenia is defined as a platelet count less than 150 × 10^9^/L, compared to severe thrombocytopenia, which is defined as a platelet count less than 75 × 10^9^/L, as mentioned in the discussion.

**Table 7:** Risk factors for treatment failure and logistic failure in the intention-to-treat population were assessed using Firth logistic regression models.

Risk Factor	Unadjusted		Adjusted	
	OR (95% CI)	*p*-Value	OR (95% CI)	*p*-Value
**Treatment Failure**				
Antimicrobial route				
IV	1 [Reference]		1 [Reference]	
Oral	1.05 (0.47–2.39)	0.901	1.38 (0.56–3.33)	0.491
Sex				
Male	1 [Reference]		1 [Reference]	
Female	0.99 (0.53–1.87)	0.986	0.99 (0.53–1.87)	0.982
Age				
Under 40	1 [Reference]		1 [Reference]	
40–59	1.52 (0.56–4.15)	0.409	1.51 (0.56–4.08)	0.410
>60 years	1.94 (0.75–4.96)	0.169	1.92 (0.76–4.89)	0.167
Peripheral vascular disease				
No	1 [Reference]		1 [Reference]	
Yes	2.80 (0.99–7.88)	0.052	2.61 (1.02–7.80)	0.045
Charlson Comorbidity Index—continuous	1.19 (1.04–1.36)	0.011	1.18 (1.04–1.36)	0.009
IVDU				
No	1 [Reference]		1 [Reference]	
Yes	0.34 (0.08–1.47)	0.149	0.36 (0.08–1.41)	0.142
Discharge Location				
Home	1 [Reference]		1 [Reference]	
Facility	1.30 (0.66–2.54)	0.446	1.29 (0.67–2.53)	0.456
Source control				
No	1 [Reference]		1 [Reference]	
Yes	0.65 (0.34–1.27)	0.211	0.66 (0.34–1.27)	0.214
Positive blood culture				
No	1 [Reference]		1 [Reference]	
Yes	1.63 (0.85–3.14)	0.143	1.04 (0.51–2.15)	0.913
Suppression therapy transition				
No	1 [Reference]		1 [Reference]	
Yes	0.68 (0.29–1.57)	0.364	0.68 (0.29–1.57)	0.356
Empiric intravenous therapy				
No	1 [Reference]		1 [Reference]	
Yes	Inestimable	Inestimable	0.32 (0.00–4.00)	0.411
**Logistic Failure**				
Antimicrobial route				
IV	1 [Reference]		1 [Reference]	
Oral	2.27 (1.00–5.15)	0.049	1.93 (0.79–4.70)	0.150
Discharge Location				
Home	1 [Reference]		1 [Reference]	
Facility	0.47 (0.22–1.00)	0.051	0.64 (0.29–1.38)	0.258
Age				
Under 40	1 [Reference]		1 [Reference]	
40–59	0.88 (0.32–2.40)	0.803	1.10 (0.40–3.09)	0.857
>60 years	0.37 (0.14–0.98)	0.045	0.46 (0.17–1.25)	0.125
Line Placed				
None	1 [Reference]		1 [Reference]	
PICC line	0.48 (0.23–1.00)	0.048	0.67 (0.30–1.54)	0.346
HD Port	0.42 (0.04–4.38)	0.469	0.89 (0.07–6.68)	0.910
Other *	0.84 (0.21–3.42)	0.810	1.44 (0.33–5.93)	0.616

Bolded headings indicate outcome categories for which logistic regression analyses were performed. IVDU = intravenous drug use. * Other lines included midlines, peripheral intravenous access, and port-a-catheters.

## Data Availability

The original contributions presented in this study are included in the article material. Further inquiries can be directed to the corresponding author(s).
